# Biochemical Markers of Bone Fragility in Patients With Diabetes

**DOI:** 10.1210/clinem/dgad255

**Published:** 2023-05-08

**Authors:** Christian Meier, Richard Eastell, Dominique D Pierroz, Nancy E Lane, Nasser Al-Daghri, Atsushi Suzuki, Nicola Napoli, Ambrish Mithal, Marlene Chakhtoura, Ghada El-Hajj Fuleihan, Serge Ferrari

**Affiliations:** Department of Endocrinology, Diabetology and Metabolism, University Hospital Basel, 4031 Basel, Switzerland; Academic Unit of Bone Metabolism, Mellanby Centre for Bone Research, University of Sheffield, S57AU Sheffield, UK; International Osteoporosis Foundation (IOF), 1260 Nyon, Switzerland; Department of Medicine and Rheumatology, Davis School of Medicine, University of California, Sacramento, CA 95817, USA; Department of Biochemistry, College of Science, King Saud University, Riyadh 11451, Saudi Arabia; Department of Endocrinology, Diabetes and Metabolism, Fujita Health University, Toyoake, Aichi 470-1192, Japan; Unit of Endocrinology and Diabetes, Department of Medicine, Università Campus Bio-Medico di Roma, 00128 Rome, Italy; Institute of Diabetes and Endocrinology, Max Healthcare, Saket, New Delhi 110017, India; Department of Internal Medicine, Division of Endocrinology, Calcium Metabolism and Osteoporosis Program, WHO Collaborating Center for Metabolic Bone Disorders, American University of Beirut Medical Center, Riad El Solh, Beirut 6044, Lebanon; Department of Internal Medicine, Division of Endocrinology, Calcium Metabolism and Osteoporosis Program, WHO Collaborating Center for Metabolic Bone Disorders, American University of Beirut Medical Center, Riad El Solh, Beirut 6044, Lebanon; Service and Laboratory of Bone Diseases, Geneva University Hospital and Faculty of Medicine, 1205 Geneva, Switzerland

**Keywords:** diabetes, bone turnover marker, sclerostin, adipokine, advanced glycation end product

## Abstract

**Context:**

The risk of fragility fractures is increased in both type 1 and type 2 diabetes. Numerous biochemical markers reflecting bone and/or glucose metabolism have been evaluated in this context.

**Objective:**

This review summarizes current data on biochemical markers in relation to bone fragility and fracture risk in diabetes.

**Methods:**

A group of experts from the International Osteoporosis Foundation and European Calcified Tissue Society reviewed the literature focusing on biochemical markers, diabetes, diabetes treatments, and bone in adults.

**Results:**

Although bone resorption and bone formation markers are low and poorly predictive of fracture risk in diabetes, osteoporosis drugs seem to change bone turnover markers (BTMs) in diabetics similarly to nondiabetics, with similar reductions in fracture risk. Several other biochemical markers related to bone and glucose metabolism have been correlated with bone mineral density and/or fracture risk in diabetes, including osteocyte-related markers such as sclerostin, glycated hemoglobin A_1c_ (HbA_1c_) and advanced glycation end products, inflammatory markers, and adipokines, as well as insulin-like growth factor-1 and calciotropic hormones.

**Conclusion:**

Several biochemical markers and hormonal levels related to bone and/or glucose metabolism have been associated with skeletal parameters in diabetes. Currently, only HbA_1c_ levels seem to provide a reliable estimate of fracture risk, while BTMs could be used to monitor the effects of antiosteoporosis therapy.

Diabetes increases the risk of fragility fracture both in type 1 (T1DM) and type 2 diabetes mellitus (T2DM), while bone mineral density (BMD) is generally low in T1DM but normal to high in T2DM compared to nondiabetics of the same sex and age. An algorithm for the identification and management of fracture risk in these patients that does not include the use of biochemical markers (1) has recently been proposed by a working group of the International Osteoporosis Foundation (IOF) on diabetes and bone. Markers of bone turnover (BTMs) have been used to further evaluate fracture risk in people affected by osteoporosis. They are mostly useful to monitor the efficacy of osteoporosis treatment, as their response is more rapid than changes in bone mass. However, biochemical markers of bone formation and resorption including osteocalcin (OC), procollagen type 1 amino-terminal propeptide (PINP), C-terminal telopeptide of type I collagen (CTX), N-terminal telopeptide of type I collagen (NTX), deoxypyridinoline, tend to be lower in diabetes patients (2), which is quite similar to what is observed in glucocorticoid-induced osteoporosis. However, their role in evaluating the severity of bone disease and predicting fracture risk in diabetes appears limited (3). In addition to the classic BTMs, other biochemical markers such as advanced glycation end products (AGEs), adipokines, cytokines, and sclerostin have been suggested to be associated with bone fragility in diabetes.

Based on a literature review by a group of experts from the IOF and European Calcified Tissue Society (ECTS) focusing on biochemical markers, diabetes, diabetes treatments, and bone in adults, we review the current evidence of the association between biochemical markers of bone fragility for the assessment of diabetic bone disease.

## Material and Methods

For this narrative review electronic literature searches were conducted through September 2022, using MEDLINE by all authors. Owing to a lack of data and limited evidence on the effects of bone markers on bone fragility in patients with diabetes, a systematic search was not feasible. The search term included all presented bone markers as well as the terms “*diabetes*,” “*type 1*,” OR “*type 2*,” “*fracture*,” OR “*bone fragility*,” OR “*bone mineral density*,” OR “*osteoporosis*,” OR “*bone quality*.” We set no language restrictions. The references were also manually searched to identify any relevant studies.

### Pathophysiology of Diabetic Bone Fragility

Extensive reviews have already been published on the differences between T1DM and T2DM (4), and more specifically on the pathophysiology of bone fragility in these metabolic disorders (5, 6). T1DM is an autoimmune disease characterized by insulin deficiency due to T-cell mediated destruction of β pancreatic cells. As the onset of T1DM usually occurs during childhood and adolescence, the accrual of bone mass is compromised resulting both in lower peak bone mass and bone mineralization caused by inflammation, insulin deficiency, and low levels of insulin-like growth factor-1 (IGF-1) (7). Meta-analyses have identified an up to 7-fold increased risk of hip fractures in patients with T1DM aged 20 to 60 years (8-10). In T2DM, the relative risk of fracture is also increased, although less dramatically (50%-100%) and after several years of disease, more so when glycemic control is poor (glycated hemoglobin A_1c_ [HbA_1c_] > 7-8%) (11, 12), eventually requires insulin, and/or microvascular complications are present. A common factor related to the chronic hyperglycemia both in T1DM and T2DM is the altered material properties of the bone tissue. The bone becomes stiffer due to an increase in covalent collagen cross-linking by pentosidine and other AGEs, and this results in a relatively hypermineralized bone (13). Hence, changes in bone matrix structure due to reduced bone remodeling and accumulation of AGEs may stiffen collagen structures, resulting in an increased susceptibility to microcracks and fragility fractures. Table 1 summarizes the striking differences between diabetes bone disease and postmenopausal osteoporosis. However, with the aging of the diabetic patient, the bone alterations due to common osteoporosis may superimpose on those induced by diabetes and thereby further contribute to their bone fragility and propensity to fractures.

**Table 1.** Differences in bone characteristics between postmenopausal osteoporosis and diabetic bone disease

Postmenopausal osteoporosis	Diabetic bone disease
− Low BMD	− Low (T1DM) or normal-high (T2DM) BMD
− Altered trabecular bone volume/structure	− Preserved trabecular bone volume/structure
− Cortical thinning and porosity	− Cortical porosity (inconsistent)
− High bone turnover	− Low bone turnover
− Lower bone mineralization	− Higher bone mineralization
− Enzymatic collagen cross-linking	− Nonenzymatic cross-linking (AGEs)
− Microcracks accumulation	− Microcracks accumulation
− Primarily driven by osteoclasts	− Primarily driven by osteoblasts and osteocytes
	− Microvascular bone disease?

Abbreviations: AGEs, advanced glycation end products; BMD, bone mineral density; T1DM, type 1 diabetes mellitus; T2DM, type 2 diabetes mellitus.

### Bone Turnover Markers

BTMs measured in serum or urine are usually classified according to the metabolic process they reflect. Bone resorption markers represent either collagen breakdown products because of osteoclastic degradation of collagenous and noncollagenous bone matrix (eg, NTX, CTX) or noncollagenous matrix proteins (eg, enzymes derived from osteoclasts like tartrate-resistant acid phosphatase 5b [TRACP]). Under normal circumstances, bone resorption is followed by the formation of new bone, which is a function of active osteoblasts. All biochemical markers of bone formation are therefore either products of collagen synthesis (eg, N- and C-terminal propeptides of type I collagen [PINP, PICP]), or proteins released by osteoblasts (eg, OC and alkaline phosphatase [ALP]) (14, 15). The introduction of automated platforms for measurement of BTMs has increased their analytical precision, but several sources of preanalytical variability (such as circadian rhythm, feeding) have to be taken into account when assessing BTMs (16). Several practical considerations need to be considered in patients with diabetes and impaired renal function. BTMs such as serum CTX, NTX, and OC are known to accumulate in advanced chronic kidney disease (CKD). Intact PINP is a useful diagnostic test, whereas total PINP is not as robust. This is because the intact PINP assay measures the trimeric PINP whereas the total PINP assay measures the trimeric and monomeric fragments of PINP. Therefore, intact PINP is the preferred assay for assessment of bone turnover in patients with diabetes and advanced renal insufficiency (17). Furthermore, it has been shown that calcitriol treatment reduces serum levels of bone turnover markers in patients with diabetes and stage 3 CKD (18).

Although elevated BTMs, specifically bone resorption markers, have been shown to be associated with increased fracture risk predominantly in postmenopausal women, their use for individual patients is of limited value (19). Hence in clinical practice BTMs are essentially useful to monitor antiresorptive or anabolic drug effects in osteoporosis and to assess compliance to therapy (20).

Based on several studies (21-28), markers of bone formation and resorption are lower in diabetic patients compared to nondiabetic controls. This has been confirmed in a recent systematic review and meta-analysis including 66 studies (62 cross-sectional, 3 randomized controlled trials, 1longitudinal study including 16-890 patients, predominantly with T2DM) confirming that diabetes is a state of low bone turnover (mean difference [95% CI]: CTX −0.10 ng/mL [−0.12 to −0.08], PINP −10.80 ng/mL [−12.83 to −8.77]) (2).

Available histological data support biochemical data of a low bone turnover state in diabetes with significant reductions in indices of bone formation (24, 25) compared to nondiabetic controls. In insulin-deficient states, such as in T1DM, this may result in delayed bone apposition during growth with microstructural alterations and ultimately increased fracture risk (29-31). In T2DM, hyperinsulinemia and insulin resistance are usually followed by decreasing β-cell capacity with insulin deficiency in longer disease duration. Hyperglycemia and insulin resistance tend to suppress bone turnover as has been observed in a cross-sectional study including many men with metabolic syndrome. In these patients, serum levels of CTX, PINP, and OC were lower compared to men without metabolic syndrome (32).

Assuming that a state of low bone turnover may be associated with microstructural alterations and bone fragility, as seen in glucocorticoid-induced osteoporosis, it would be tempting to use BTMs as a marker of fracture risk in diabetes. In fact, several studies observed an association with BTMs and prevalent fracture risk. Jiajue et al (33) have shown that suppressed bone turnover (as defined by low levels of PINP) was associated with increased fracture risk in nonobese postmenopausal Chinese women with T2DM, independent of BMD. This finding was consistent with the study by Yamamoto et al (34), who documented an inverse relation between decreased serum levels of OC (as well as low parathyroid hormone [PTH] levels) and an increased risk of prevalent vertebral fractures (VFs). The study by Jiajue and colleagues (33), however, showed that BTMs may be differently related to prevalent fracture risk as serum levels of CTX were positively associated with fracture occurrence.

The relationship between BTMs and incident fractures in T2DM has recently been investigated in 2 studies (35). Neither longitudinal study showed an association between BTMs and fracture risk. This was true for the Peking Vertebral Fracture Study including 26 postmenopausal women with T2DM and incident VFs and non-VFs in which serum levels of CTX and PINP were assessed (35). Based on the Health ABC Study, Napoli et al (3) confirmed these observations in a larger cohort of 169 diabetic and 172 prediabetic adults with T2DM (mean follow-up time for clinical fracture of any type, 7.0 ± 2.9 years). There were no significant associations between BTM levels (CTX, OC, and PINP) and the risk of incident vertebral and wrist fractures. Hence, this indicates BTMs may not be suitable to predict fracture risk in T2DM.

No randomized controlled trials evaluated the efficacy of osteoporosis drugs in diabetic patients. Post hoc analyses predominantly investigated the effect on BMD and fracture risk. It might be speculated that antiresorptive treatments may have a detrimental effect on bone quality due to reduction of preexisting low bone turnover. Nevertheless, a population-based study by Vestergaard et al (36) confirmed that further reduction in bone turnover by antiresorptive drugs does not result in an increased fracture risk in diabetics. Antiresorptive drugs seem to change BTMs in diabetics similarly to nondiabetics (37). This has been confirmed in a recent study based on a data set of individual patient data from randomized, placebo-controlled trials of osteoporosis therapies (SABRE project). Based on a meta-regression approach, it has been shown that antiresorptive drugs appear to be equally effective in those with and without T2DM with similar changes in BMD (after 24 months) and bone marker levels (3-12 months) and fracture risk reduction. Concerns regarding possible reduced antifracture efficacy in T2DM due to lower BTMs in T2DM were not borne out (38). Therefore, it can be postulated that for the evaluation of treatment compliance and efficacy changes in BTMs are of similar magnitude as known from trials assessing treatment effects in postmenopausal osteoporosis.

### Effects of Diabetes Medications on Bone Markers

#### Metformin

Most of the data come from randomized studies using metformin as a control or in add-on to insulin treatment. In the ADOPT trial (39), CTX declined by 1.3% in women and 12.7% in men in the metformin group similar to PINP (−14.4% and −19.3% in women and men, respectively). Data from the South Danish Diabetes Study showed a lower level of bone formation without concomitant reduction in bone resorption when metformin was combined with insulin (40). In a recent post hoc analysis, PINP increased less in the metformin + insulin compared to the placebo + insulin group (*P* = .001), while the increases in CTX levels were not statistically different (41). In summary, the reported effect of metformin on BTMs in humans is variable and still inconclusive.

#### Sulfonylureas

In the ADOPT trial mentioned earlier, CTX was reduced by 3.3% in women and 4.3% in men, while change in PINP and bone-specific alkaline phosphatase (BSAP) were only marginal for glyburide (39). In Japanese patients urinary NTX excretion in postmenopausal women treated with sulfonylurea was significantly lower (42). Thus, while preclinical studies suggest a positive effect of sulfonylureas on bone formation, evidence from clinical studies indicates that sulfonylureas do not affect bone turnover significantly in humans.

#### Thiazolidinediones

Most, but not all, studies have shown an increase in bone resorption markers like CTX with the use of rosiglitazone, while the effects on bone formation markers have been variable (39, 43-47). Pioglitazone has shown conflicting results with regard to the effects on BTMs. Following 3 months of treatment with pioglitazone, serum concentrations of PINP and BSAP were significantly decreased from baseline in women, but not in men (48). A metanalysis showed minimal changes in BTMs in thiazolidinedione users compared to nonusers. Pooled summary statistics showed differences in CTX of 11.0% (*P* = .04), BSAP of 1.0% (*P* = .80), PINP of 3.7% (*P* = .40), and in OC of −.8% (*P* = .70) (49). Reasons for the heterogeneity remain unclear.

#### Incretin-based therapies

##### Dipeptidyl peptidase-4 inhibitors

In a study comparing metformin and sitagliptin in postmenopausal diabetic women, serum ALP was significantly decreased, serum OC levels were nonsignificantly decreased, while urinary deoxypyridinoline decreased significantly in the sitagliptin treated group (50). Whether the effect of dipeptidyl peptidase-4 inhibitors on BTMs may be molecule specific and not a class effect needs to be clarified.

##### Glucagon-like peptide-1 receptor agonists

No significant change in BTMs despite weight loss and improvement in glycemic control have been found with exenatide (51, 52). In a recent, randomized, double-blinded clinical trial, liraglutide treatment for 26 weeks did not have an effect on bone resorption marker CTX (53). Overall, studies suggest that glucagon-like peptide-1 receptor agonists may have minor, insignificant effects on BTMs in the clinical setting.

#### Sodium glucose co-transporter 2 inhibitors

Canagliflozin increases serum CTX in a dose-dependent manner, which is significantly correlated with a reduction in body weight. It also increases serum OC levels (54). No significant changes in PINP were seen with canagliflozin treatment (55). Dapagliflozin treatment did not result in significant changes in markers of bone formation or resorption at week 50 (56) or week 102 (57). The VERTIS MET trial showed that mean serum CTX at week 26 was increased in the ertugliflozin groups in a dose-related manner, but mean PINP and PTH levels were similar to baseline (58). In pooled data analyses, no changes in either serum ALP levels or urinary NTX/creatinine ratio were observed with empagliflozin (59, 60).

The effect of sodium glucose cotransporter 2 (SGLT2) inhibitors on BTMs seems to be minor, with subtle differences between agents. The effect on bone turnover is unlikely to explain effects of SGLT2 inhibitors on fracture risk, if any.

In summary, BTMs appear to be relatively insensitive to the effects of glucose-lowering drugs and their changes are therefore unlikely to reflect antidiabetic drug effects on fracture risk. Table 2 summarizes the effects of these antidiabetic drugs on different parameters.

**Table 2.** Effects of antidiabetic drugs on bone turnover, bone mineral density, and fracture risk (6, 61)

	Bone turnover marker		BMD	Fracture risk
	Bone formation	Bone resorption		
Metformin	↓/∼	↓/∼	∼/↑	↓/∼
Sulfonylureas	↑/∼	↓/∼	ND	↓/∼/↑
Thiazolidinediones	↓↓/∼/↑	↑↑/∼	↓↓/∼	↑↑
GLP-1 analogues	∼	↓↓	↑/∼	∼
DPP4 inhibitors	↓/∼	∼	∼	∼/↓
SGLT2 inhibitors	∼	∼/↑	∼	∼/↑
Insulin	∼	∼	∼	↑

Abbreviations: ↑∣increased; ↓∣decreased; ∼∣unchanged; BMD, bone mineral density; DPP4, dipeptidyl peptidase inhibitor 4; GLP1, glucagon-like peptide 1; ND, not determined; SGLT2, sodium/glucose cotransporter 2.

### Glycemic Markers

Hyperglycemia seems to play an important role in the regulation of bone turnover. However, few studies have investigated the effect of glucose in relation BTMs. Most studies have not been powered for this outcome and little evidence has been confirmed.

Hyperglycemia has been shown in studies to decrease osteoclast and osteoblast activity (62, 63) in vitro. Along these lines, the meta-analysis by Hygum et al (2) identified hyperglycemia to be a significant contributor to the differences in OC and sclerostin in patients with T1DM and to the differences in PINP, OC, NTX, and sclerostin in patients with T2DM compared with controls. Furthermore, with improvement in glycemic control, there is an increase in OC in some but not all studies (64-68).

In a Danish cross-sectional study, Starup-Linde et al (69) found that each 5-mmol/L increase in glucose was associated with CTX, PINP, and total OC reductions of 10%, 8%, and 6%, respectively. The strongest effect was observed for undercarboxylated OC, which was reduced by 38%. However, a sensitivity analysis confirmed a significant effect in T1DM only for CTX and PINP, whereas none of the aforementioned markers were shown to be associated with serum glucose in T2DM. When authors tested the effect of HbA_1c_, they found a significant, negative association, only with OC. A recent study including patients with T1DM confirmed a significant association between HbA_1c_ levels and lower bone formation as indicated by lower PINP levels (70).

It should be highlighted that a single nonfasting glucose level is a poor measure of glucose metabolism in diabetics that, mostly in T1DM, is not considered useful in clinical practice. The low correlations between HbA_1c_ and most of the investigated markers may indicate a weak association between glucose control and bone turnover.

Using isoglycemic intravenous glucose infusion (IIGI) in healthy male participants, no significant associations at any time points with OC or undercarboxylated OC levels have been found (71). Another important study underscores the evidence that glucose per se does not have a direct role on BTMs but the effect may be mediated by production of incretin hormones (72). Administrating either an oral glucose tolerance test or an IIGI to healthy male participants induced a 50% reduction in serum CTX, while an approximately 30% reduction was reached by IIGI. PINP was not influenced by either intervention. A linear regression analysis revealed that peak gastric inhibitory peptide significantly predicted nadir serum CTX, and that peak gastric inhibitory peptide could explain 34% of the variability in nadir CTX (72).

An important clinical question is to what extent glycemic control might affect the risk of fracture. For patients with T1DM, only a few studies have investigated the association between the degree of glycemic control and fracture risk. While Forsen et al (73) reported a nonsignificant trend between glycemic control and fracture risk, Neumann et al (74) observed a significant relationship between serum HbA_1c_ levels and the risk of any clinical fracture. The odds ratio (OR) for each 1-SD increase in median HbA_1c_ was 1.92 (95% CI, 1.09-2.75). A recent nested case-control study based on a primary care database (UK-based Clinical Practice Research Datalink) including 3329 patients with T1DM confirmed these findings (12). The risk of incident nonvertebral low-trauma fractures was slightly increased in patients with 3-year mean HbA_1c_ levels greater than 8% compared with patients with HbA_1c_ levels of 7% or less (OR 1.39; 95% CI, 1.06-1.83).

Data in patients with T2DM remain inconclusive, with studies reporting an association between glycemic control and the risk of fracture (75-77) whereas others failed to confirm this relationship (12, 73, 78, 79). In the Rotterdam Study, fracture risk was increased in patients with HbA_1c_ levels greater than 7.5% compared to those with better glycemic control (hazard ratio [HR] 1.62; 95% CI, 1.09-2.40) (77). In a large geriatric population with T2DM, Li et al (75) observed a U-shaped relationship between HbA_1c_ levels and fracture risk with increased risk both in patients with elevated HbA_1c_ levels of 9% to 10% and in those with low HbA_1c_ levels of less than 6% compared with HbA_1c_ levels of 6% to 7% (HR 1.24; 95% CI, 1.02-1.49 and HR 1.19; 95% CI, .97-1.45, respectively). Of note and according to a recent meta-analysis and regression, the positive association between low HbA1c levels and the risk of fracture appears to be, in part, explained by hypoglycemia-induced falls, possibly related to insulin use (11).

### Advanced Glycation End Products

AGEs and their receptor (RAGE) are considered to play important roles in diabetes-related inflammation, which results in glucose intolerance and diabetic complications (80-82). Hyperglycemia as well as the polyol pathway and lipid peroxidation lead to increased production of AGEs, the products of nonenzymatic glycation of macromolecules. AGEs can promote RAGE expression on cell membranes, and the AGE-RAGE interaction elicits intracellular signaling system, and the extracellular secreted form of soluble RAGE (sRAGE) acts as decoy receptor for AGE (83). AGE/RAGE production have been reported to be risk factors for osteoporosis (84, 85) and sRAGE could prevent AGE-RAGE interactions. Therefore, low serum endogenous sRAGE level has been reported to be associated with increased risk of VF in T2DM (86). However, sRAGE is not always a useful marker for assessment of the AGE-RAGE pathway because it can increase in either excess RAGE production or destruction (87).

AGEs physically affect the properties of the bone materials, especially through accumulation in the bone collagen fibers (88). Posttranslational modification of collagen is crucial for collagen stability, and it contributes to bone strength. Among several biomarkers for glyco-oxidation, pentosidine is an intermolecular cross-linker for AGEs and can be used as a surrogate marker of total AGE formation (88, 89). Impaired enzymatic cross-linking and an increase in nonenzymatic cross-links like pentosidine in bone have been proposed as major cause of bone fragility in aging, osteoporosis, and diabetes. Higher baseline levels of urinary pentosidine in osteoporosis patients are potential risk factors for incident VFs when these patients are treated with bisphosphonates (90). Bone pentosidine content estimated by bone biopsy in T1DM was higher in patients with fracture than without fracture (13). Cross-sectional and prospective studies found serum and urinary pentosidine levels are associated both with vertebral and clinical fractures in nondiabetes and diabetes patients (86, 90-94). Nevertheless, whether pentosidine could be considered as a valuable biochemical marker to guide patient care in diabetic bone disease remains to be established (95). Of note, there are noninvasive options available to measure AGEs in the skin with an AGE-reader, which appears to be more reflective of long-term AGE accumulation. A relation between skin AGEs and fractures has been shown in the general population (96).

### Osteocyte-related Markers

#### Receptor activator for nuclear factor kappa-B-ligand and osteoprotegerin

Receptor activator for nuclear factor kappa-B-ligand (RANKL) produced by osteocytes and osteoblasts, in addition to bone marrow T cells, is an essential factor for the differentiation and activation of osteoclast (97, 98). Osteoprotegerin (OPG) acts as a soluble decoy receptor for RANKL. OPG is produced by the osteoblast lineage, including osteocytes, and is also released from vascular endothelial cells in response to inflammatory stimuli, suggesting its role in both bone metabolism and cardiovascular calcification (99). There are several studies showing an association between serum OPG levels and BMD (100-103). However, the precise role of serum OPG level for the assessment of osteoporosis and fracture risk has not yet been elucidated.

Transient changes of RANKL and OPG expression by glucose have been shown in osteocytes but the direct effect of glucose was not so apparent as on sclerostin (104). On the contrary, AGEs increased RANKL and sclerostin in osteocyte-like cells, and induced apoptosis of these cells (105). Serum OPG levels are reported to be higher both in T1DM and T2DM (106, 107), which could reflect an inhibition of bone resorption (and turnover) in bone, and also be a biomarker for microvascular complications and macrovascular disease in diabetes (108, 109).

#### Sclerostin

Sclerostin is secreted by osteocytes and inhibits osteoblast activity as part of bone's adaptative response to mechanical loading (110). Serum sclerostin has been positively associated with fasting blood glucose and declines in response to acute hyperinsulinemia (111, 112). On the contrary, fasting blood glucose level or glucose variability did not contribute to serum sclerostin level, while other BTMs such as serum CTX and PINP were significantly affected by blood glucose levels (113). A study also showed no significant difference in sclerostin levels across different HbA_1c_ levels (114). However, a recent meta-analysis concluded that serum sclerostin is higher both in T1DM and T2DM (2). Changes in serum sclerostin levels were significantly correlated with changes in serum CTX levels in pioglitazone-treated men, but not in the metformin-treated diabetics (115).

Taken together with recent evidence of higher sclerostin gene expression in bone of diabetic individuals (116), those observations suggest a possible involvement of sclerostin in low bone turnover in diabetes. In addition, serum sclerostin could be a predictive marker for cardiovascular risk in these patients (117, 118).

### Proinflammatory Cytokines

Hyperglycemia is associated with increased levels of both circulating and localized proinflammatory cytokines measured from clinical tissues and clinical studies that include tumor necrosis factor α (TNFα), interleukin (IL)-1, IL-6, and IL-18. These inflammatory markers have been reported in the early years of T1DM, and they may influence the accrual of peak bone mass in adolescents and young adults (119-123). Among T2DM patients, an inverse association has been observed between BMD and TNFα and IL-6 (124). Interestingly, diabetics are less able to downregulate localized inflammation compared to age-similar controls.

The role of inflammation with T2DM is less clear and may be mediated indirectly. Many individuals with T2DM are obese, and adipocytes can activate inflammation by producing reactive oxygen species (ROS) that then increase the production of inflammatory cytokines that can reduce osteoblast number through apoptosis and simultaneously stimulate osteoclastic bone resorption. Interestingly, this process becomes constant as ROS stimulate the mesenchymal stem cells to preferentially differentiate into adipocytes rather than into osteoblasts, resulting in reduction in the transcription of Wnt proteins to further inhibit bone formation. Also, activation of RAGE that is expressed on osteoblasts can increase both inflammatory cytokines and ROS and continue the activate bone remodeling and bone loss.

Interestingly, a recent study of T2DM individuals in Japan found C-reactive protein (CRP), a marker of systemic inflammation, was associated with incident bone fractures in these patients even after adjustment for BMD, previous fracture, and age. As CRP production is driven by IL-6, this study suggests a link between systemic inflammation in T2DM patients and bone fragility (125). Based on these data, it appears that inflammation, either directly or indirectly through activation of AGEs, and RAGE, alters bone remodeling, and over time this reduces bone strength.

### Vascular Markers

Diabetes is often discussed as a state of accelerated aging and the pro-inflammatory cytokines that are associated with both T1DM and T2DM may accelerate both microvascular and macrovascular disease (126-128). Preclinical studies in diabetic mice have reported that cytokine expression of proangiogenic factors, especially vascular endothelial growth factor (VEGF), by bone marrow cells is significantly decreased and endothelial cells show reduced responsiveness to proangiogenic cytokines and well as a reduced ability to form vascular tubules in vitro (129, 130). This can result in bone marrow cell apoptosis, which is observed in T2DM. However, currently, there are no known vascular markers that are associated with bone changes with diabetic patients.

### Adipokines

*Adipokines*, or *adipocytokines*, is a collective term for peptide hormones released in the adipose tissue. Since the discovery of the first adipokine (leptin), the perception of adipose tissue as a basic storage depot for fats has been transformed to what is known today as an endocrine organ responsible for many metabolic processes in the human body (131). These endocrine functions of several adipokines were suggested to link obesity to most of the chronic noncommunicable diseases since it facilitates crosstalk between different cells not only within the adipose tissue but to other organs in maintaining overall energy homeostasis (132). While the exact mechanisms in the crosstalk between fat, bone, and muscle tissues are still emerging, the unifying theme of chronic, low-grade inflammation and how altered levels of adipokines affect these tissues may be central in understanding their collective contribution in the overlapping progression of chronic conditions such as β-cell dysfunction in diabetes (133), osteoporosis (6), and sarcopenia (134). Biomarkers are measured more commonly using multiplex assays since they are studied more frequently in clusters and simultaneously rather than in isolation. Table 3 summarizes some of the major adipokines studied for their role in bone fragility in individuals with T2DM.

**Table 3.** List of adipokines and inflammation markers and its observed association with bone mineral density in the type 2 diabetes mellitus population

Adipokine	BMD	T2DM population	Reference
**Leptin**	+	Men (N = 28) and women (N = 12)	Tamura et al, 2007 (135)
		2-y postbariatric surgery patients (N = 54)	Maghrabi et al, 2015 (136)
		Men (N = 168)	Vasilkova et al, 2011 (137)
	—	Men (N = 93) and women (N = 89)	Kurajoh et al, 2019 (138)
**Adiponectin** Total and/or HMW	-	Men (N = 42) and women (N = 38)	Lenchik et al, 2003 (139)
		Men (N = 231)	Kanazawa et al, 2009 (140)
		Men (N = 207) and women (N = 272)	Register et al, 2013 (141)
		With osteoporosis (N = 80)	Chen et al, 2017 (142)
		Women with osteoporosis (N = 90)	Al-Osami and Hameed 2018 (143)
	+	Men (N = 32) (total only)	Kanazawa et al, 2010 (144)
**RBP4**	+	Men (N = 109) and women (N = 165)	Huang et al, 2018 (145)
**TNFα and IL-6**	—	With osteoporosis and diabetic nephropathy (N = 76)	Zhao 2018 (124)

(+) denotes positive association; (−) denotes inverse association.

Abbreviations: BMD, bone mineral density; HMW, high-molecular-weight; IL-6, interleukin-6; RBP4, retinol-binding protein 4; T2DM, type 2 diabetes mellitus; TNFα, tumor necrosis factor α.

#### Leptin

Leptin is a 167-amino acid protein primarily described to be associated with appetite regulation and energy balance (146). Leptin resistance is thought to occur among obese individuals, and elevated levels of leptin negatively influences β-cell function through several mechanisms (133). Furthermore, leptin has been observed to be a potent inhibitor of OC through a hypothalamic relay and regulation of sympathetic signals brought to osteoblasts, thereby stimulating osteoblast production (147, 148). But studies also indicate that leptin, via its action on the central nervous system and the β-adrenergic system, is catabolic to vertebral trabecular bone by increasing RANKL expression in osteoblasts (149). A few studies have reported positive association between leptin levels and BMD, independent of body mass index (BMI) in people with T2DM (136-137), but not all (138).

#### Adiponectin

Adiponectin is a 30-kDa collagen-like adipokine mostly known for its antiatherogenic and insulin-sensitizing functions. Low circulating levels of adiponectin have been a consistent hallmark feature of endothelial dysfunction and insulin resistance (150). Most observational studies have consistently shown an inverse association between adiponectin and BMD (143-141) in individuals with T2DM. Among the roster of adipokines, elevated circulating levels of adiponectin have been implicated as an independent risk factor for fracture, at least in older men (151), and progressive bone loss in women (152). While the mentioned studies used nondiabetic populations, these observations highlight not only the clinical effect of adiponectin in bone health but that its effects are also sexually dimorphic.

#### Retinol binding protein 4

Retinol binding protein 4 (RBP4) is an adipokine that belongs to the lipocalin family with major functions in retinol transport and serves as a biomarker for glucose metabolism and vascular injury secondary to insulin resistance (153, 154). Positive associations between RBP4 and BMD have been reported both in T2DM (145) and non-T2DM populations, the latter of which included postmenopausal women with osteoporosis (155). Cumulatively, these limited studies suggest RBP4's protective role in bone maintenance similar to other known protective factors such as BMI and weight.

The associations of key adipokines such as leptin, adiponectin, RBP4, as well as inflammatory factors such as TNFα and IL-6, with BMD among individuals with T2DM highlight the need for further investigations and the inclusion of other adipokines in relation to fracture risk among diabetics.

### Hormones

#### Insulin and insulin-like growth factor-1

Insulin anabolic actions on osteoblasts appear to be modulated by the IGF-1 receptor (156). In vitro studies have revealed that hyperglycemia or AGEs may cause osteoblast resistance to the anabolic effects of IGF-1 (157, 158). Patients with T1DM have β–cell failure and low levels of insulin and IGF-1, and this reduces osteoblastogenesis (5). Case-control studies have revealed substantial and consistent reductions in IGF-1 levels in adolescents and young adults with T1DM (159-161). Associations between poor glycemic control and IGF-1 levels were found in one study (160), but not another (159). The mechanism by which insulin resistance affects IGF-1 molecular signaling at the level of osteoblast in T2DM is less clear (6).

A few studies examined the association between IGF-1 and BMD parameters (162-164), hip quadrant analysis (159, 165), fractures (162, 164, 166, 167), and fracture risk. There was a positive correlation between IGF-1 and BMD at various sites in adults with T1DM and T2DM (162-164). However, one study in men and women with T2DM showed in a multivariate analysis adjusting for age, duration of diabetes, BMI, serum creatinine, and HbA_1c_ that IGF-1 was not a significant predictor of BMD (164). Furthermore, a recent cross-sectional study from China of 391 patients with T2DM evaluated the association of IGF-1 with BMD in each sex separately (168). In men, IGF-1 was a positive predictor of hip but not spine BMD on multivariate analyses, whereas it was not in women (168). In men with T1DM, IGF-1 correlated with femoral neck superoanterior quadrant cortical thickness and superoposterior quadrant average trabecular volumetric BMD, evaluated using quantitative computed tomography, possibly contributing to increased bone fragility (165). Conversely, in another study in young women with T1DM, compared to age-matched controls, IGF-1 was not significantly associated with the apparent trabecular spacing measured by magnetic resonance imaging (159). Of note, the mean age at diagnosis of T1DM was 22.6 years for the former study (165), while it was 9 years in the latter one (159), and this might be the cause of a discrepancy in findings compared to other studies.

Four studies assessed the correlation of IGF-1 with non-VFs, identified through interviews (167), and morphometric VFs, identified through thoracic and lumbar spine x-ray (162, 164, 166, 167). In a cross-sectional study involving 582 men and 412 postmenopausal women with T2DM for more than 20 years, only in women was a higher IGF-1 associated with a lower risk of one or more VFs (166). In particular, the OR of VFs was reduced by 30% per SD increase in IGF-1 level (166). Similar results were not reproduced in men (164, 166). However, both in men and women, combining IGF-1 levels and lumbar spine or femoral neck BMD predicted the risk of VF better than either parameter alone (166). The results on VFs were replicated in a study of 482 postmenopausal women with long-standing diabetes (162). Higher IGF-1 was associated with a lower risk of VF, with a stronger association with increasing number of fractures (1 VF [OR = 0.58; *P* = .041], 2 VFs [OR = 0.42; *P* = .012], and ≥3 VFs [OR = 0.19; *P* = .001] in the T2DM group). Recently, a mendelian randomization study confirmed that IGF-1 levels are associated with a decreased overall fracture risk, an effect that remained after adjustments for height (169).

#### Calciotropic hormones

Alterations in the calcium-sensing-vitamin-D–PTH axis have been reported both in T1DM and T2DM. Osmotic diuresis due to glycosuria may induce a state of enhanced excretion of calcium and magnesium through the tight coupling of calcium and magnesium handling at the medullary thick ascending loop, resulting in a negative calcium and magnesium balance (6). It has been suggested that impaired calcium-sensing, possibly due to hypomagnesemia, may cause a state of functional hypoparathyroidism, potentially contributing to low bone remodeling (170).

In patients with T1DM, serum mean 25-hydroxyvitamin D (25(OH)D) level, an index of vitamin D nutritional status, was lower (159, 161), and mean PTH level higher (161), or similar (159, 171), compared to controls. In a study of 250 patients with T1DM and 250 age- and sex-matched controls, PTH levels were higher in patients than controls, and there was a significant negative correlation between serum 25(OH)D and HbA_1c_ levels (172). Some evidence suggests that hyperparathyroidism has an adverse effect on glucose metabolism (173). Conversely, there was no difference in mean serum 25(OH)D and PTH levels between tertiles of HbA_1c_ in 94 adult patients with long-standing T1DM (174). The aforementioned studies did not provide serum creatinine levels as a potential cofounder of high PTH, although individuals with CKD were excluded.

In contrast to T1DM, PTH levels were more consistently low in patients with T2DM (34, 175, 176), and correlated with lower levels of BTMs (176). Serum 25(OH)D levels (and OC levels), but not PTH levels, differed between the 3 categories of glycemic control in a cohort of 240 patients with T2DM, levels being higher with lower HbA_1c_ (177). In a study of 480 individuals with T2DM, PTH levels were not associated with insulin levels, indices of glycemic control, nor of insulin resistance (178). Higher serum levels of 25(OH)D and BTMs were associated with a lower risk of metabolic bone disease in a cross-sectional study of 2671 adults partaking in a 6-month population-based study in Pomerania (179).

Ten studies in T2DM patients showed an inconsistent association between 25(OH)D and PTH level and BMD at various sites (138, 180-188). One study of 785 patients with T2DM showed that PTH, but not 25(OH)D level, is a significant predictor of BMD at the lumbar spine, femoral neck, and total hip (186). The same study examined the predictors of major osteoporotic fracture and hip fracture risk using FRAX, and similarly showed that PTH, but not 25(OH)D level, was positively correlated both with major osteoporotic fracture and hip fracture risk (186). In another cross-sectional study of 182 participants with long-standing T2DM (mean duration 10 years) PTH was not a significant predictor of trabecular BMD nor cortical thickness using high-resolution peripheral quantitative computed tomography (138).

Two studies evaluated PTH level and fracture risk and found conflicting results, most likely related to different patient profiles and sample size (34, 189).

In summary, the correlation between BMD and hormones, IGF-1, PTH, and 25(OH)D, in diabetic patients is variable and does not allow a definite conclusion. The available data in women suggest that increased IGF-1 levels in women with T2DM are associated with a reduced risk of VFs and non-VFs. Conversely, studies on fracture risk in association with low calciotropic hormones are scarce and suggest a possible increased risk of VFs, but need to be confirmed in further research.

### Clinical Implications and Future Directions on the Use of Biochemical Markers in Diabetic Bone Disease

Biochemical markers play an important role in the management of osteoporosis, but their role in diabetic bone disease, both in T1DM and T2DM, is less clear. So far, the most convincing biomarker of fracture risk in diabetes appears to be HbA_1c_ itself, which recapitulates the fact that poorly controlled and complicated diabetes is the main determinant of bone fragility and fracture risk. As a consequence, better glycemic control, and thereby prevention of microvascular complications, eventually with drugs that also have a direct favorable activity on bone cells (such as metformin), remains the primary approach to prevent impairment of bone quality in patients with diabetes. In addition, it would be usual practice to measure 25(OH)D and PTH levels, and if vitamin D deficiency is found, to provide adequate vitamin D repletion.

Whereas in postmenopausal women high BTMs are associated with an increase in fracture risk, in diabetes, the level of BTMs is low and not consistently predictive of fractures, so they should not be interpreted as an indication of low fracture risk in these patients. However, in patients with diabetes and low BMD and/or fragility fractures in whom antiosteoporotic drugs such as bisphosphonates are started, BTMs remain useful to monitor drug compliance and perhaps efficacy (1). It might be speculated that antiresorptive treatments may have a detrimental effect on bone quality due to reduction of preexisting low bone turnover. Nevertheless, a recent meta-analysis confirmed that further reduction in bone turnover by antiresorptive drugs does not result in an increased, but rather decreased, fracture risk in diabetics.

Notwithstanding their currently limited clinical use in this context (Table 4), the study of biomarkers has been helpful in better understanding the pathogenesis of diabetic bone disease, particularly the importance of low bone turnover, as this has turned our attention to alterations in bone quality, such as changes in bone matrix collagen properties and mineralization. One of the most promising biochemical markers is AGEs, which not only affect collagen cross-linking but also potentially the activity and perhaps viability of osteoblast lineage cells. AGEs have been associated with fracture risk in diabetes, but larger and prospective studies are needed to further evaluate their potential use in fracture prediction. This may also be true regarding other biochemical markers such as proinflammatory cytokines, adipokines, and other markers discussed here, particularly IGF-1. Among them, sclerostin seems to be a promising circulating marker of diabetic bone disease, since it might reflect not only the level of osteocyte dysfunction and suppression of bone formation that occurs in this disease, but also potentially the vascular alterations that themselves are associated with specific bone alterations such as cortical porosity (190). Unfortunately, there are several different sclerostin assays available with inconclusive results regarding the relationship between circulating sclerostin and parameters of bone frailty (191).

**Table 4.** Serum markers with potential role for fracture risk assessment in diabetes

Marker	Tissue of origin	Role in diabetic bone	Potential use for fracture prediction
Glycemia (HbA_1c_)	Erythrocytes	Collagen glycosylation Fracture risk prediction	Yes (fracture data)
Bone turnover marker (PINP, CTX)	Collagen formation/degradation	State of low turnover Evaluation of treatment efficacy	No predictive value
AGEs (pentosidine)	Collagen cross-linking	Impair bone properties by accumulation in bone collagen	Potentially (limited fracture data)
Sclerostin	Osteocyte	Higher sclerostin gene expression in DM	Potentially (no fracture data)
IGF-1	Hepatocyte	Osteoblast stimulation	Yes (fracture data)
Calciotropic hormones (eg, PTH)	Parathyroid cells	Calcium homeostasis	Potentially (limited fracture data)
Proinflammatory cytokines (eg, TNFα, IL-1, -6, -11, CRP)	Inflammation	Alteration of bone remodeling	Potentially (limited fracture data)
Vascular markers (eg, VEGF)	Bone marrow	Bone marrow cell apoptosis	No fracture data
Adipokines (eg, leptin, adiponectin)	Adipose tissue	Regulation of osteoblast function	No fracture data

Abbreviations: AGEs, advanced glycation end products; CRP, C-reactive protein; CTX, C-terminal telopeptide of type I collagen; DM, diabetes mellitus; HbA_1c_, glycated hemoglobin A_1c_; IGF-1, insulin-like growth factor-1; IL, interleukin; PINP, procollagen type 1 amino-terminal propeptide; PTH, parathyroid hormone; TNFα, tumor necrosis factor α; VEGF, vascular endothelial growth factor.

Future research is needed to further understand the role of biochemical markers in the evaluation of diabetic bone disease. Specifically, the role of bone markers to predict fracture risk needs further investigation. So far only glycemic control, AGEs, and serum IGF-1 seem to have potential for fracture prediction in patients with diabetes. Other biomarkers, such as periostin or periostin fragment, which have been associated with bone microstructure and fragility fractures (192), or circulating dipeptidyl peptidase inhibitor 4, potentially associated with vascular disease in diabetes, are under investigation.

## Data Availability

Data sharing is not applicable to this article as no data sets were generated or analyzed during the current study.

## Abbreviations

25(OH)D: 25-hydroxyvitamin D
AGEs: advanced glycation end products
ALP: alkaline phosphatase
BMD: bone mineral density
BMI: body mass index
BSAP: bone-specific alkaline phosphatase
BTM: bone turnover marker
CKD: chronic kidney disease
CRP: C-reactive protein
CTX: C-terminal telopeptide of type I collagen
DM: diabetes mellitus
ECTS: European Calcified Tissue Society
HbA_1c_: glycated hemoglobin A_1c_
IGF-1: insulin-like growth factor-1
IIGI: isoglycemic intravenous glucose infusion
IL: interleukin
IOF: International Osteoporosis Foundation
NTX: N-terminal telopeptide of type I collagen
OC: osteocalcin
OPG: osteoprotegerin
PINP: procollagen type 1 amino-terminal propeptide
PTH: parathyroid hormone
RAGE: AGE receptor
RANKL: receptor activator for nuclear factor kappa-B-ligand
RBP4: retinol binding protein 4
ROS: reactive oxygen species
SGLT2: sodium glucose cotransporter 2
sRAGE: soluble RAGE
T1DM: type 1 diabetes mellitus
T2DM: type 2 diabetes mellitus
TNFα: tumor necrosis factor α
VEGF: vascular endothelial growth factor
VF: vertebral fracture
